# Aspects diagnostiques, thérapeutiques et pronostiques du diabète gestationnel au Centre Hospitalier Universitaire Sylvanus Olympio

**DOI:** 10.11604/pamj.2019.34.18.18917

**Published:** 2019-09-10

**Authors:** Kodjo Agbeko Djagadou, Toyi Tchamdja, Komi Dzidzonu Némi, Abago Balaka, Mohaman Awalou Djibril

**Affiliations:** 1 Service de Médecine Interne, Faculté des Sciences de la Santé, Université de Lomé, Lomé, Togo; 2 Service de Médecine Interne, Faculté des Sciences de la Santé, Université de Kara, Kara, Togo

**Keywords:** Diabète gestationnel, diagnostic, complications, Lomé, Gestational diabetes, diagnostic, complications, Lomé

## Abstract

**Introduction:**

Les objectifs de cette étude étaient de décrire les aspects diagnostiques, pronostiques et thérapeutiques du diabète gestationnel au CHU Sylvanus Olympio de Lomé.

**Méthodes:**

Il s'est agi d'une étude descriptive transversale réalisée sur 5 ans allant du 1^er^ janvier 2013 au 31 décembre 2017. Elle a concerné 125 gestantes ayant accouché, suivies en médecine interne et dans le Service de Gynéco-obstétrique.

**Résultats:**

La moyenne d'âge maternelle était de 30,84±4,17 ans. Les facteurs de risque les plus rencontrés étaient le surpoids et l'obésité (57,7%), antécédent de diabète familial (33,3%), antécédent de fausse couche spontanée (26,6%), antécédent de mort fœtale in utero (MFIU) (15,5%) et de l'antécédent du diabète gestationnel (8,8%). Le dépistage du diabète gestationnel a été réalisé par la glycémie à jeun et hyperglycémie provoquée (HGPO) à 75g. Le diagnostic a été posé au premier trimestre dans 55,6% des cas, au deuxième trimestre dans 33,3% et 11,1% au troisième trimestre. Le recours à l'insulinothérapie a été nécessaire dans 24,4% des cas et 66,6% sous régime seul. Soixante-six virgule sept pour cent (66,7%) des femmes ont accouché par césarienne et 33,3% par voie basse. Parmi les complications maternelles à l'accouchement, nous avons retrouvé 22,2% de HTA, 17,7% de pré-éclampsie et 2,2% de RPM. Parmi les complications du nouveau-né, il y avait 48,9% de macrosomie, 11,1% de prématurité, 11,1% d'hypoglycémie, 4,4% de malformation et 4,4% mort-né. Quatre-vingt-huit virgule neuf pour cent (88,9%) des nouveaux nés avaient un score d'APGAR supérieur à 7 et plus de 48% étaient des macrosomes.

**Conclusion:**

Le diabète gestationnel entraîne des complications materno-fœtales. La nécessité d'un dépistage systématique est obligatoire même en l'absence de facteur de risque afin de programmer une prise en charge optimale.

## Abstract (en)

**Introduction:**

The purpose of this study was to describe the diagnostic, prognostic and therapeutic features of gestational diabetes at the Sylvanus Olympio University Hospital Center.

**Methods:**

We conducted a retrospective descriptive study over a period of 5 years from 1^st^ January 2013 to 31^st^ December 2017. It involved 125 pregnant women who had given birth in the Department of Internal Medicine and in the Gyneco-Obstetric Department.

**Results:**

The average age of patients was 30.84±4.17 years. The most common risk factors were overweight and obesity (57.7%), a family history of diabetes (33.3%), a history of spontaneous miscarriage (26.6%), a history of fetal death in utero (15.5%) and a history of gestational diabetes (8.8%). Gestational diabetes screening was performed using fasting blood glucose test and 75g oral glucose-tolerance test. Diagnosis was made in the first quarter in 55.6% of cases, in the second quarter in 33.3% and in the third quarter in 11.1%. Insulin therapy was necessary in 24.4% of cases and a healthy meal-plan alone in 66.6%. Sixty-six point seven percent (66.7%) of women had given birth by cesarean section and 33.3% vaginally. Maternal complications at birth included: arterial hypertension (22.2%), preeclampsia (17.7%) and premature ruptured membranes (2.2%). Newborn complications included macrosomia (48.9%), prematurity (11.1%), hypoglycaemia (11.1%), malformations (4.4%) and stillborn child (4.4%). Eighty-eight point nine percent (88.9%) of newborns had Apgar score greater than 7 and more than 48% were macrosomes.

**Conclusion:**

Gestational diabetes leads to maternofetal complications. Systematic screening is essential even in the absence of risk factors for optimal patient management.

## Introduction

Le diabète gestationnel (DG) est un trouble de la tolérance glucidique, de sévérité variable, diagnostiqué pour la première fois pendant la grossesse, quelle qu'en soit l'étiologie, l'ancienneté et l'évolution après la grossesse [1]. La prévalence du diabète gestationnel est très variable selon les populations étudiées [2]. Chez les femmes australiennes, cette prévalence est de 4,3%, contre 15% chez les femmes nées dans le sous-continent indien [3]. En Afrique subsaharienne, les prévalences rapportées sont plus fortes. Au Sénégal, la prévalence par rapport au nombre de cas de grossesse était de 33,1% en 2015 [4]. En Côte d'Ivoire elle était de 38,8% en 2008 [5]. Le diabète gestationnel représente un problème de santé publique dans le monde, du fait de sa fréquence qui a considérablement augmenté et de son retentissement maternel et fœtal [4]. Les complications du diabète gestationnel sont redoutables et s'observent à court, moyen et long terme justifiant une prise en charge optimale multidisciplinaire [3]. Elles sont dominées chez la mère par l'hypertension artérielle gravidique et la pré-éclampsie dont leur fréquence est doublée par rapport à la population générale, passant respectivement de 3,3% à 7,3% et de 3,9% à 8% [5]. Chez le fœtus on peut noter le risque de retard de croissance in-utero, de mort in-utero et de macrosomie fœtale [6]. Cette étude a été initiée au Centre Hospitalier Universitaire Sylvanus Olympio de Lomé afin de déterminer la fréquence du diabète gestationnel, d'identifier ses facteurs de risque, de déterminer ses circonstances de découverte, de déterminer ses complications materno-fœtales et de décrire sa prise en charge thérapeutique.

## Méthodes

### Cadre et méthodes d'étude

Notre étude s'était déroulée dans les Services de Médecine Interne et de Gynéco-obstétrique du Centre Hospitalier Universitaire Sylvanus Olympio. Nous avons réalisé une étude rétrospective descriptive sur une durée de 4 ans. Tous les dossiers comportant la mention diabète gestationnel et une hyperglycémie provoquée par voie orale (HGPO) à 75g étaient inclus. Les données collectées comprenaient les données épidémiologiques (age, statut socioprofessionnel, antécédents obstétricaux (gestité, parité), facteurs de risque observés chez nos patientes, les données cliniques (indice de masse corporelle calculé à partir du poids habituel avant la grossesse et de la taille, circonstances de découverte du diabète gestationnel) et les données para-cliniques (glycémie, bilan lipidique, l'ECBU, hémoglobine glyquée, bilan rénal, échographie obstétricale). Les données pronostiques (complications materno-fœtales, les modalités d'accouchement) et les données thérapeutiques (régime, insulinothérapie) avaient été également étudiés. La saisie et le traitement des données ont été faites par le logiciel RStudio version 3.4.2., les logiciels Microsoft Excel 2007 et Microsoft Word 2007.

## Résultats

### Caractéristiques socio-démographiques

Nous avons colligé 125 cas de diabète gestationnel du 1^er^ janvier 2014 au 31 décembre 2017 soit une période de 4 ans, période au cours de laquelle il y a eu 40579 accouchements avec pour fréquence 0,3%. Les tranches d'âge les plus représentées étaient celle de 30 à 34 ans et celle de 35 à 39 ans dans respectivement 40% (n = 50) et 28,88% (n = 36) des cas. L'âge moyen des femmes était 30,87±4,17 ans avec des extrêmes de 23 ans et 37 ans. Les salariées étaient les plus représentées dans 68,8% (n = 86).

### Aspect diagnostique

#### Circonstances de découverte

Les prescriptions diagnostiques ou de dépistage du DG étaient réalisées selon les facteurs de risque représentés dans le Tableau 1. La suspicion diagnostique était faite par une glycémie à jeûn et la confirmation était obtenue par HGPO à 75g. Les facteurs statistiquement associés à la prescription d’un dépistage étaient: un antécédent de diabète gestationnel (p < 0,002), l’IMC (p < 0,003), un antécédent de macrosomie (p = 0,002) et un antécédent familial de diabète (p < 0,001). Une prise de poids de plus de 12kg et l'âge maternel n’étaient pas statistiquement associés à une prescription de dépistage dans notre étude. Le Tableau 2 nous montre la répartition des gestantes selon le terme de découverte du diabète gestationnel.

**Tableau 1:** Répartition des gestantes selon des facteurs de risque

	n	(%)
Surpoids	53	42,2
Obésité	51	40,8
Antécédent de diabète familial	42	33,6
Antécédent de diabète gestationnel	44	35,2
Antécédent de mort fœtal in utéro	19	15,2
Antécédent de fausse couche spontanée	33	26,4
Antécédent de macrosomie	19	15,2

**Tableau 2:** Répartition des gestantes selon le terme de la grossesse

	N	%
16-20 Semaines d’aménorrhée	69	55,2
24-28 Semaines d’aménorrhée	45	36
32-36 Semaines d’aménorrhée	11	8,8

#### Aspect thérapeutique

Le régime alimentaire du diabétique était prescris seul dans 75,2% (n = 94) des cas. Dans 24,8% (n = 31) des cas, ce régime seul était insuffisant pour normaliser la glycémie et était donc associé à l'insulinothérapie.

### Aspect pronostique

#### Comorbidités maternelles

Les gestantes ont présenté plusieurs comorbidités notamment l'hypertension artérielle dans 22,4% des cas (n = 28), la pré-éclampsie dans 8% des cas (n = 10) et la rupture prématurée des membranes dans 2,4% des cas (n = 3). La plupart des gestantes ont accouchées par voie haute dans 66,4% des cas (n = 83). L'indication prépondérante de la césarienne était la macrosomie fœtale dans 56% des cas (n = 70). Les nouveaux nés étaient à terme (37 SA - 40 SA + 6 Jours) dans 75,2% des cas (n = 94) et la prématurité représentait 10,4% (n = 13) des cas. La vitalité fœtale était satisfaisante avec un score d'apgar supérieur à 7 dans 88% des cas (n = 110). Selon le poids de naissance, les macrosomes et les hypotrophes représentaient respectivement 48,8% (n = 61) et 10,4% (n = 13) des cas. L'hypoglycémie à la naissance était notifiée chez 45 nouveaux nés. Cinq mort-nés étaient notés.

#### Suivi dans le post-partum

La glycémie s'était spontanément normalisée chez 68 femmes. Dix femmes étaient devenues des diabétiques de type 2. Les autres gestantes ont été perdue de vue.

## Discussion

La fréquence du diabète gestationnel est très diversement appréciée dans la littérature, elle varie de 1% à 17% selon les études occidentales et asiatiques [7] et de 5% à 38% selon les études africaines [8]. Notre fréquence faible de 0,3% pourrait s'expliquer non seulement par le fait que certaines patientes ayant des facteurs de risque aient pu échapper au dépistage et par l'exclusion des dossiers ne comportent par HGPO.

Le surpoids et l'obésité étaient les facteurs de risque les plus rencontrés dans notre étude. En effet le surpoids et l'obésité constituent l'un des facteurs de risque majeur du diabète gestationnel [9]. Ils aggravent l'insulino-résistance physiologique qui apparaît au cours du deuxième et troisième trimestre de grossesse secondaire à une sécrétion de l'hormone lactogène placentaire par le placenta surtout chez les femmes génétiquement prédisposées [2]. La plupart des auteurs sont unanimes sur leur prédominance mais leur fréquence est diversement rapportée selon les séries [10]. Par conséquent, les femmes en âge de procréer doivent lutter contre le surpoids, l'obésité et la sédentarité qui constituent les facteurs de risque évitables du diabète gestationnel. Le dépistage ciblé sur les facteurs de risque a été quasiment la circonstance de découverte des cas de diabète gestationnel. Toutes nos patientes ont été dépistées par la glycémie à jeun et confirmées par HGPO à 75g selon la méthode de l'OMS [11]. Cette méthode de dépistage serait la plus facile, la mieux adaptée et économiquement réalisable dans nos régions.

Dans notre série, le diabète gestationnel était associé à l'HTA, à la pré-éclampsie et à la macrosomie. Cette association serait multifactorielle car il est de plus en plus admis que l'élévation du risque d'HTA gravidique et de pré-éclampsie pourrait avoir comme facteur le diabète gestationnel et l'indice de masse corporelle. La césarienne a été en majorité le mode d'accouchement dans notre série dans 66,4% (n = 83) des cas. Ce qui pourrait être liée au taux élevé de la pré-éclampsie et de la macrosomie.

Chez la plupart des femmes atteintes du diabète gestationnel, la tolérance au glucose revient à des niveaux normaux après l'accouchement [1]. Certaines femmes continuent toutes fois d'afficher une glycémie élevée. De nombreux auteurs recommandent de poursuivre l'auto-surveillance glycémique après l'accouchement [1]. Chez nos patientes des glycémies à jeun normales ont été obtenues dans le post-partum. Cependant plusieurs patientes ont été perdues. Ce qui explique que l'HGPO à 75g n'a pas été pratiquée à six mois après l'accouchement. Les femmes chez qui le diabète a disparu après la grossesse doivent faire l'objet d'un dépistage au tout début de la grossesse suivante car le taux de réapparition du diabète gestationnel étant d'environ 67% [1].

## Conclusion

A travers cette étude, nous avons constaté que le diabète gestationnel est bien fréquent à Lomé. La majorité des femmes présentait les facteurs de risque comme l'obésité et le surpoids. Le DG est pourvoyeur de grossesses à risque de complications materno-fœtales. Le dépistage doit être suffisamment précoce et systématisé par l'épreuve d'hyperglycémie provoquée par voie orale pour que la prévention puisse être efficace. Une standardisation du suivi par une équipe multidisciplinaire (gynécologue-obstétricien, interniste, diabétologue, nutritionniste, sage-femme et néonatalogiste) est nécessaire. Par ailleurs, il est important de sensibiliser les gestantes à la nécessité d'un suivi métabolique post-partum puisque ces femmes présentent un risque accru d'hyperglycémie ultérieure.

### Etat des connaissances actuelles sur le sujet

- Le diabète gestationnel représente un problème de santé publique dans le monde, du fait de sa fréquence qui a considérablement augmenté et de son retentissement maternel et fœtal tels que l'hypertension artérielle gravidique, la pré-éclampsie, le retard de croissance in-utero, de mort in-utero et de macrosomie fœtale.

### Contribution de notre étude à la connaissance

- Le diabète gestationnel est pourvoyeuse de grossesses à risque de complications materno-fœtales. Le dépistage doit être suffisamment précoce et systématisé par l'épreuve d'hyperglycémie provoquée par voie orale pour que la prévention puisse être efficace.

## Conflits d’intérêts

Les auteurs ne déclarent aucun conflit intérêts.
